# An Experimental Study on the Dynamic Mechanical Properties of Epoxy Polymer Concrete under Ultraviolet Aging

**DOI:** 10.3390/ma14082074

**Published:** 2021-04-20

**Authors:** Yutian Liao, Dongpeng Ma, Yiping Liu, Zhenyu Jiang, Zejia Liu, Licheng Zhou, Liqun Tang

**Affiliations:** School of Civil Engineering and Transportation, State Key Laboratory of Subtropical Building Science, South China University of Technology, Guangzhou 510640, China; 201720100022@mail.scut.edu.cn (Y.L.); 201610101013@mail.scut.edu.cn (D.M.); zjliu@scut.edu.cn (Z.L.); ctlczhou@scut.edu.cn (L.Z.); lqtang@scut.edu.cn (L.T.)

**Keywords:** dynamic behavior, epoxy polymer concrete (EPC), hygrothermal conditions, Split Hopkinson Pressure Bar (SHPB), ultraviolet aging

## Abstract

Epoxy polymer concrete (EPC) is widely applied in engineering for its excellent mechanical properties. The impact loads and severe climatic conditions such as ultraviolet radiation, temperature change and rain erosion are in general for its engineering practice, potentially degrading the performance of EPC. In this paper, a procedure of accelerated aging for EPC, imitating the aging effect of ultraviolet radiation and hygrothermal conditions based on the meteorological statistics of Guangzhou city, was designed. After various periods of accelerated aging, the dynamic behaviors of EPC were studied by using a Split Hopkinson Pressure Bar (SHPB). The verification of the experimental data was performed. The two-stage dynamic compression stress-strain curves were obtained: (a) linear growth stage following by strain hardening stage at impact velocity 12.2 m/s and 18.8 m/s, (b) linear growth stage and then a horizontal stage when impact velocity is 25.0 m/s, (c) linear growth stage following by strain softening stage at impact velocity 29.2 m/s. The experimental results show that the specimens after longer accelerated aging tend to be more easily broken, especially at impact velocity 12.2 m/s and 18.8 m/s, while the strain rate is the main factor affecting the compression strength and stiffness. Ultimately the influence of strain rate and equivalent aging time on dynamic increase factor was revealed by a fitting surface.

## 1. Introduction

Epoxy Polymer Concrete (EPC) is a new type of concrete material, which is mainly made of sand and gravel as aggregate and epoxy resin matrix as binder. With the advantages of short curing time and excellent mechanical properties [1,2], it has become one of the commonly used materials for road and bridge pavement [2,3], expansion joint construction [4,5], railway sleepers [6,7,8], construction reinforcement [9,10,11] and other engineering practices. When used in practical application, EPC is not only exposed to ultraviolet (UV) radiation, along with high temperature and humidity in the natural environment, but also experienced typical dynamic loads such as explosion and vibration. Therefore, it is important to investigate the dynamic mechanical properties of EPC after aging in UV radiation and hygrothermal conditions.

Until now, many researches have been conducted on EPC, including its quasi-static mechanical properties such as bending and compressive strengths. Also, possible solutions to optimize the performance of EPC such as improving the rubber-to-stone ratio [12,13] and adding reinforcing materials [14,15,16,17] have been proposed. Environmental hazards such as ultraviolet radiation, temperature and chemical media will weaken the performance of epoxy resin-based composite materials [18,19]. Researches carried out by Reis et al. [20,21,22] showed that UV and temperature decrease the fracture toughness and tensile strength of the EPC. The studies of Hassani et al. [23] and Oussama et al. [24] both showed that the strength of EPC decreases at a temperature of 250 °C. In addition, the degradation effect of thermal cycling on the fracture toughness and tensile strength of EPC were also investigated [25]. When exposed to chemical media such as acid, the strength of EPC decreases more significantly [9,26]. Ma et al. [27] analyzed the effect of an environment with strong ultraviolet radiation, high temperature and high humidity on the bending performance, and the experimental results showed that 4 years of the equivalent accelerated aging reduce 8.4% of the EPC’s bending strength.

The dynamic mechanical properties of concrete materials have attracted much attention, and the Split Hopkinson Pressure Bar (SHPB) has been used as a common testing device to evaluate materials’ impact performance. The strain rate sensitivity is proven to be one of the significant properties of concrete [28,29,30,31,32,33]. In recent years, some researchers found that severe environmental factors would seriously deteriorate the dynamic performance of concrete [34,35,36,37]. Unfortunately, few studies focus on the influence of aging effects on the dynamic behaviors of EPC.

Unlike previous studies, the aim of our study was to test the dynamic properties of EPC after UV radiation and the hygrothermal cycle by use of a SHPB device. Considering EPC’s applications in pavement and fortification, which are not only exposed to the natural environment but also experience typical dynamic loads, the accelerated aging scheme was designed to imitate the natural conditions of South China based on the meteorological data in Guangzhou, and the dynamic response was investigated under impact loading. The cylindrical EPC specimens were prepared to undergo five different accelerated aging durations; four different impact velocities were adopted, and at least four specimens, in which three specimens to got a repetition and another extra just in case, were tested under the same accelerated aging time and strain-rate. The dynamic compression stress-strain curves with impact velocity ranged from 12.2 m/s to 29.2 m/s and different equivalent aging time ranging from 0 years to 6 years were obtained. The failure phenomenon was analyzed to reveal the degradation of EPC after aging. The strain rate sensitivity of EPC was studied and the relationship between dynamic increase factor and strain rate and accelerated aging time was described by a fitting surface. The contribution of this research is the broadening of current knowledge of dynamic performance of EPC after aging. Although it is a basic research, it is favorable for the application and deterioration evaluation of EPC in engineering.

The structure of the paper is as follows: in Section 2, the preparation of EPC is presented. Next, an experiment scheme is designed to study the dynamic properties of EPC after accelerated aging. In Section 3, the validation of the experimental data is discussed. Then the characteristic of dynamic responses is analyzed and the failure phenomenon under different impact velocities and equivalent aging time is investigated. Subsequently, a discussion of the strain rate sensitivity of EPC is presented. Finally, a fitting surface revealing the relationship between dynamic increase factor and strain rate and accelerated aging time is constructed. Section 4 provides the main conclusions summarizing the most significant achievements of the article.

## 2. Specimen Preparation and Testing Program

### 2.1. Materials

**Figure 1:**
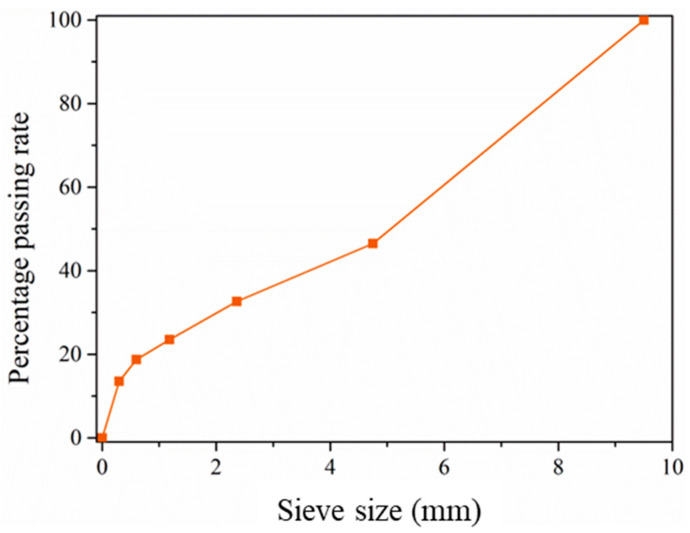
Percentage passing rate of aggregate.

The EPC specimens herein were made by mixing epoxy resin, amine hardener and granite aggregate. The granite aggregate was provided by Fujian Shiyufa Stone Co., Ltd. The density of aggregate is 2.63 g/cm^3^ and the gradation is shown in Figure 1. Bisphenol-A epoxy resin and amine hardener were provided by Fuzhou Baisheng Fine Chemicals Pte. Ltd. The density and Epoxy Equivalent Weight (EEW) of the resin were 1.1 g/cm^3^ and 200 g/equiv, respectively. The density and Amine Hydrogen Equivalent Weight (AHEW) of the hardener were 0.985 g/cm^3^ and 100 g/equiv respectively.

### 2.2. Specimen Preparation

**Figure 2:**
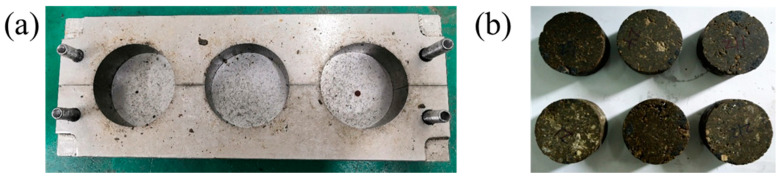
(**a**) Metal mold and (**b**) specimen after demolding.

The procedure of the preparation of the EPC specimens is as follows. Primarily that the materials were all mixed by hand, and after drying in a 40 °C oven for 4 h, aggregates were mixed with epoxy resin and hardener thoroughly according to the mass ratio of 100:8.7:4.3, i.e., the weight percentage of resin is 11.5 wt.% and the ratio of epoxy resin and hardener is 2:1. The proportion is close to the recommended one, which ensures performance and at the same time minimizes cost [38]. Then, the mixture was filled into the metal mold as shown in Figure 2a and demolded after curing for 3 days at 25 °C. The properties of EPC proved to be constant after 3 days curing [39]. The mixing process and the density of the specimens were strictly controlled to guarantee the uniformity of the specimens, in which high voids content due to under-compaction or crushed aggregates caused by over-compaction were avoided. The cylindrical specimens were 35mm high with a diameter of 70 mm, as shown in Figure 2b. The ratio of height to diameter was set to 1:2 to ensure that the time to achieve uniform stress state in SHPB experiment was short enough.

### 2.3. SHPB Device and Parameters

**Figure 3:**
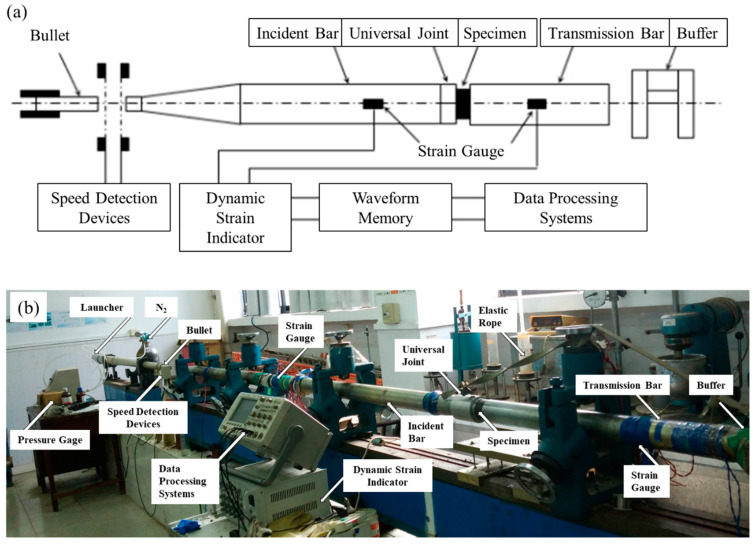
Right cone SHPB system: (**a**) schematic diagram, (**b**) photo.

The impact compression tests were carried out on a right cone SHPB system as shown in Figure 3. The bullet and bars were all made of steel, and the impact velocity of the bullet was controlled by a triggered air pressure. The diameters of the bullet and the front end of incident bar were both 37 mm, and the diameters of the rear end of incident bar and the transmission bar were both 74 mm. The length of the bullet, incident bar (including the cone) and transmission bar were 60 cm, 270 cm and 200 cm respectively; The distance between the strain gauge and the rear end of incident bar was 150 cm, and the distance between the strain gauge on the transmission bar and contact surface with specimen was 70 cm.

### 2.4. Accelerated Aging Equipment and Parameters

The accelerated aging tests of EPC specimens were carried out on the GB-UV-B ultraviolet light weather test chamber produced by Guangzhou Zhenyu Climate Environment Testing Equipment Co., Ltd. There were 8 lamps in the test box; each emitted ultraviolet light with a power of 12 W. Concerning the average distance between the lamps and the specimen was 70 mm, the intensity of ultraviolet light acting on the specimen’s surface on average was 1560 W/m^2^ [28]. The ultraviolet radiant quantity of South China is 262.4 MJ/m^2^ per year. To imitate the ultraviolet radiant quantity, the accelerated aging time corresponding to natural aging for one year was 93.3 h, calculated by [27]

(1) $$t=\frac{262.4\times 10^{6}}{1560}\times 2\;s=93.3\;h$$

**Figure 4:**
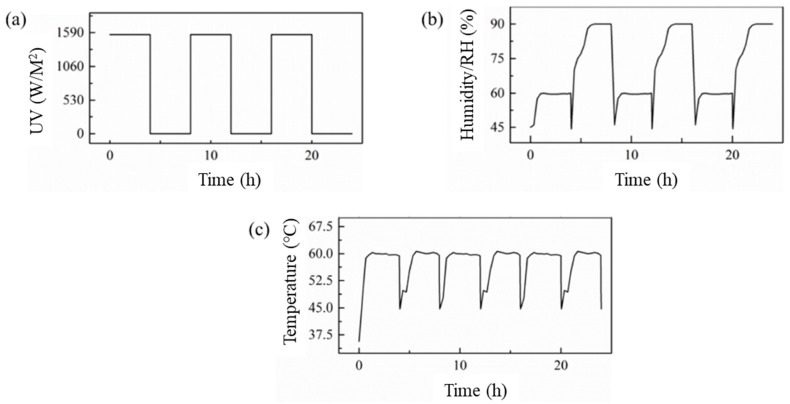
Time history curve of parameters: (**a**) UV-light, (**b**) temperature, (**c**) humidity.

In the accelerated aging test, the natural diurnal variation was simulated by the periodic alternation of UV radiation and condensation process with equal duration. The time of the periodic alternation was 8 h, and the changes of ultraviolet radiation, temperature and humidity with time are shown in Figure 4. Standard Practice for Operating Fluorescent Ultraviolet Lamp Apparatus for Exposure of Nonmetallic Materials (ASTM G 154-16) [40] was used to design the aging procedure. The relative humidity (RH) was set to 90% to simulate the humidity in South China. When the temperature decreases, the spray accumulates into drops and the humidity decreases from 90% to 60%. In this paper, the EPC specimens underwent equivalent aging for 1 year, 2 years, 4 years and 6 years with a blank control group without aging.

## 3. Experimental Results and Discussion

### 3.1. Validation of the Experimental Data

In the SHPB experiment, the stress wave is assumed to be one-dimensional, and the stress is assumed to be uniformly distributed in the cross-section of the specimen. The relationship of the compression stress σ(t), (Pa), compression strain ε(t), and strain rate $\dot{\varepsilon }(t)$, (s^−1^), are as follows

(2) $$\sigma (t)=\frac{A_{B}E_{B}}{2A_{S}}[\varepsilon_{t}(t)+\varepsilon_{r}(t)+\varepsilon_{i}(t)]$$

(3) $$\varepsilon (t)=\frac{C_{B}}{L_{S}}\int_{0}^{t}[\varepsilon_{t}(t)+\varepsilon_{r}(t)-\varepsilon_{i}(t)]d\tau$$

(4) $$\dot{\varepsilon }(t)=\frac{C_{B}}{L_{S}}[\varepsilon_{t}(t)+\varepsilon_{r}(t)-\varepsilon_{i}(t)]$$

where $A_{B}$, (m^2^), $E_{B}$, (Pa) and $C_{B}$, (m/s) are the cross-section area, elastic modulus and elastic wave velocity of the bar, respectively. $L_{S}$, (m) and $A_{S}$, (m^2^) are the length and cross-section area of the specimen, respectively. $\varepsilon_{i}(t)$, $\varepsilon_{t}(t)$ and $\varepsilon_{r}(t)$ are the incident, transmission and reflection strains, respectively. It is worth mentioning that all the variables discussed in this paper are derived from the impact compression experiment, and that means the elastic modulus here is referred to as the compression elastic modulus; the same is true of the other variables.

As we know, the distribution of aggregates and micro pores in EPC is not uniform, and the wave impedance of EPC specimens is relatively small, which may affect the stress uniformity in the specimen. To evaluate whether the stress in the specimen reaches a uniform state when the stress wave reaches the interface at the *k*th time, the stress disturbance parameter $\alpha_{k}$ is defined as

(5) $$\alpha_{k}=\frac{\Delta \sigma_{k}}{\sigma_{k}}$$

where $\sigma_{k}$, (Pa) is the stress at the interface, and $\Delta \sigma_{k}$, (Pa) is the disturbance caused by the transmission wave as well as the reflection wave at the interface.

**Figure 5:**
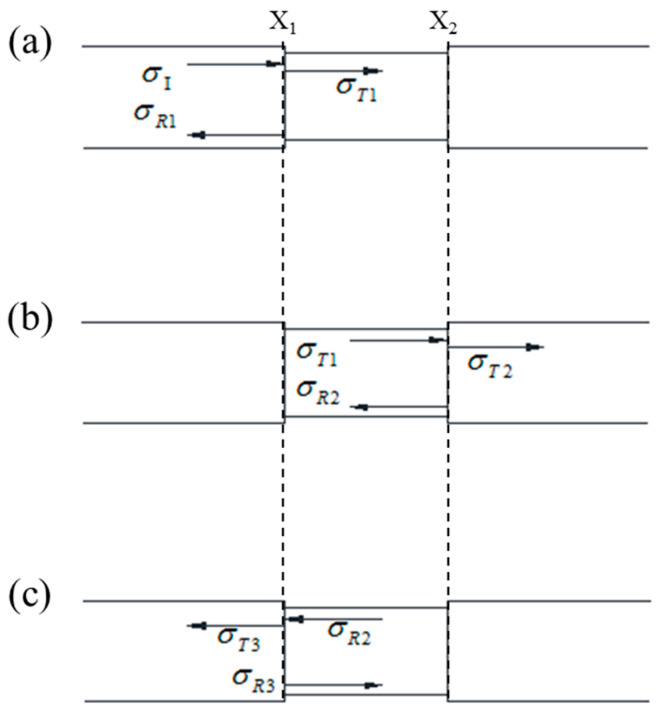
Reflection and transmission of elastic wave at specimen interface: (**a**) $t<\tau_{S}$, (**b**) $\tau_{S}<t<2\tau_{S}$, (**c**) $2\tau_{S}<t<3\tau_{S}$.

The elastic wave velocity in the bar and the specimen are $C_{B}=\sqrt{\frac{E_{B}}{\rho_{B}}}$ and $C_{S}=\sqrt{\frac{E_{S}}{\rho_{S}}}$, respectively, where $\rho_{B}$, (kg/m^3^) and $E_{B}$, (Pa) are the density and the elastic modulus of the bar; $\rho_{S}$, (kg/m^3^) and $E_{S}$, (Pa) are the density and the elastic modulus of the specimen. Obviously, the wave impedance of the bar and the specimen are $\rho_{B}C_{B}$ and $\rho_{S}C_{S}$, respectively. When the stress wave transfers from the bar to the specimen, the reflection coefficient is defined as $R_{1}=\frac{1-n_{BS}}{1+n_{BS}}$, and the transmission coefficient is defined as $T_{1}=\frac{2}{1+n_{BS}}$, where $n_{BS}$ is the ratio of the generalized elastic wave impedance between the bar and the specimen expressed as $n_{BS}=\frac{(\rho_{B}C_{B})A_{B}}{(\rho_{S}C_{S})A_{S}}$. Similarly, when the stress wave transfers from the specimen to the bar, we have $R_{2}=\frac{1-n_{SB}}{1+n_{SB}}$ and $T_{2}=\frac{2}{1+n_{SB}}$, where $n_{BS}$ is ratio of the generalized elastic wave impedance between the specimen and the bar expressed as $n_{SB}=\frac{(\rho_{S}C_{S})A_{S}}{(\rho_{B}C_{B})A_{B}}$. The process of the stress wave entering the specimen is shown in Figure 5. Taking the time when the incident wave reaches the interface as t=0, the interval of stress wave propagating through specimen $\tau_{S}$, (s) is expressed as $\tau_{S}=\frac{L_{S}}{C_{S}}$.

When $t<\tau_{S}$, the stress wave is transmitted into the specimen at the interface X_1_ where the incident bar and the specimen contact, the stress wave disturbance is $\Delta \sigma_{1}=\sigma_{T1}$, and $\sigma_{T1}=T_{1}\sigma_{I}$ is the stress wave transmit from the bar to the specimen. 

When $\tau_{S}<t<2\tau_{S}$, the stress wave is reflected into the specimen at the interface X_2_ where the transmission bar and the specimen contact, and the stress wave disturbance equals $\Delta \sigma_{2}=R_{2}\sigma_{T1}=R_{2}\Delta \sigma_{1}$.

When $2\tau_{S}<t<3\tau_{S}$, the stress wave is reflected into the specimen again at the interface X_1_, the stress wave disturbance equals $\Delta \sigma_{3}=R_{2}^{2}\sigma_{T1}=R_{{}_{2}}^{2}\Delta \sigma_{1}$.

Similarly, when the stress wave reaches the interface for the *k*th time, the disturbance of the stress wave can be calculated as follows

(6) $$\Delta \sigma_{k}=R_{{}_{2}}^{\left(k-1\right)}\Delta \sigma_{1}$$

And the stress at the interface after the *k*th’s transmission and reflection can be written as

(7) $$\begin{array}{c} \sigma_{k}=\sum_{i=1}^{k}\Delta \sigma_{i}\\ =\frac{1-R_{2}^{k}}{1-R_{2}}T_{1}\sigma_{I}\\ =(1-R_{2}^{k})\sigma_{I} \end{array}$$

Substituting Equations (5) and (6) into Equation (4), we obtain

(8) $$\alpha_{k}=\frac{R_{{}_{2}}^{\left(k-1\right)}(1-R_{2})}{1-R_{{}_{2}}^{k}}$$

As we know, the stress and strain in the specimen are considered uniform approximately when $\alpha_{k}\leq 5\%$. In this study, the velocity of the stress wave in the specimen is about 3715 m/s, and the thickness of the specimen is 35 mm. The density and elastic compression modulus of the specimen and bar are shown in Table 1. Substituting the values into Equation (7), we have k≥7, which means only when the stress wave is reflected at least seven times can the stress in the specimen be considered as uniform, i.e., the stress wave should travel back and forth three times before the stress achieve uniform in the specimen. The corresponding time is about 55 μs. 

**Figure 6:**
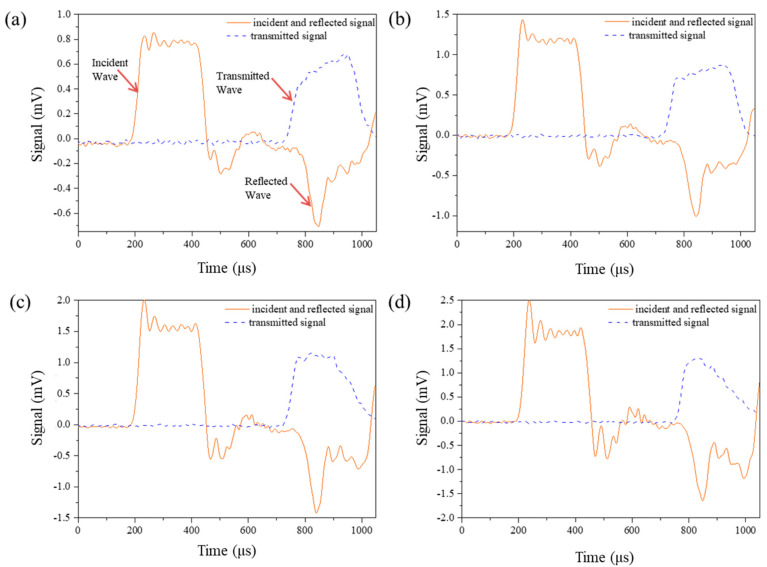
Pulse signal with different impact velocities: (**a**) 12.2 m/s, (**b**) 18.8 m/s, (**c**) 25.0 m/s, (**d**) 29.2 m/s.

Table 2 shows the relationship between impact velocity and trigger pressure, and Figure 6 shows the typical pulse signal under different impact velocities. The loading time of the incident wave is about 270 μs at all impact velocities. It can be found in the transmission waves that the time when failure occurred are all about 200 μs after the first reflection, much longer than the required 55 μs. Therefore, the specimen has enough time to achieve the stress uniformity before failure, which ensures the validity of the experimental results.

### 3.2. Compression Stress-Strain Curves and Failure Phenomenon

**Figure 7:**
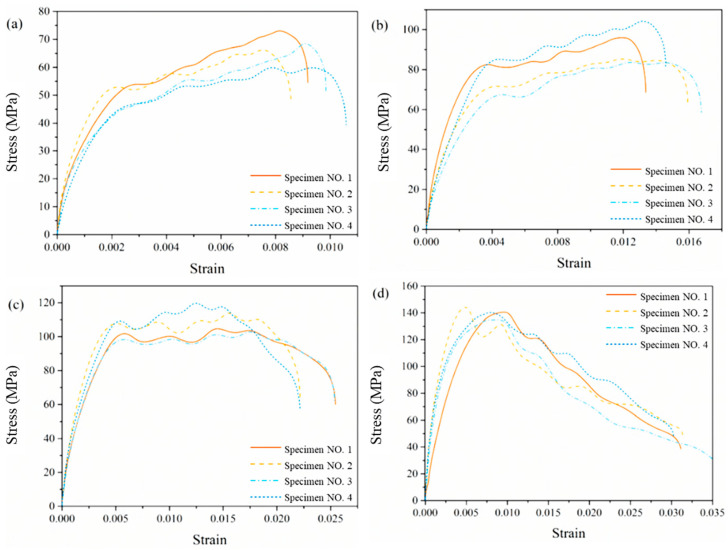
Compression Stress-strain curves of specimens without aging treatment under different impact velocities: (**a**) 12.2 m/s, (**b**) 18.8 m/s, (**c**) 25.0 m/s, (**d**) 29.2 m/s.

Figure 7 shows the compression stress-strain curves of the blank control group without aging treatment. The experimental results of specimens in the same group have good repeatability. Obviously, the compression stress-strain curves share the same morphological characteristics, including similar characteristic values such as the slope of the linear growth stage, the time when the first peak appears, and etc., as shown in Figure 7. Therefore, the average compression stress-strain curves under the same impact velocity and aging time are used to analyze in the following discussion.

**Figure 8:**
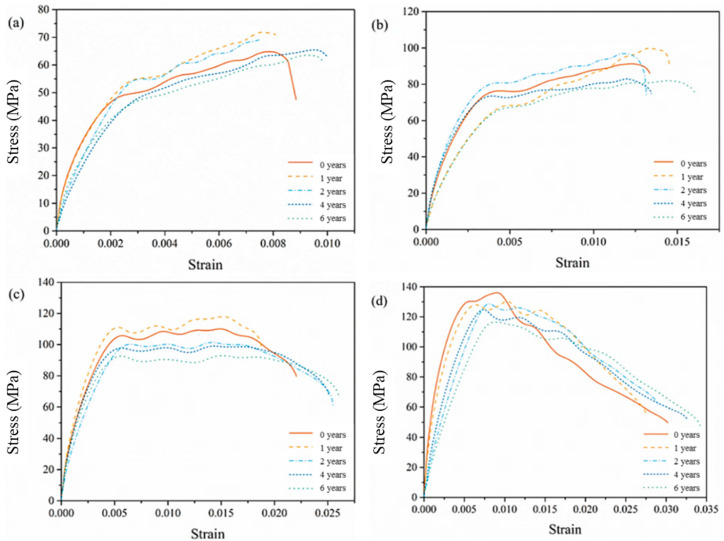
Average compression stress-strain curves with different equivalent aging time under different impact velocities: (**a**) 12.2 m/s, (**b**) 18.8 m/s, (**c**) 25.0 m/s, (**d**) 29.2 m/s.

Average compression stress-strain curves with different equivalent aging time under different velocities are shown in Figure 8. All the curves show obvious two-stage form regardless of aging time. However, with different impact velocities, the features of the curves are various. When the impact velocity is relatively low, such as 12.2 m/s and 18.8 m/s, the curves grow linearly, following by strain hardening stage. The demarcation stress of the two stages is roughly 50 MPa and 80 MPa, respectively. When the impact velocity reaches 29.2 m/s, a strain softening phenomena are obvious after the stresses achieve a certain peak value. In the case of impact velocity 25.0 m/s, it seems to be a property transforming speed and the second stages of the curves are nearly horizontal with no obvious strengthening or softening effects. This is similar to the experimental results of dynamic compression tests of unsaturated polyester polymer concrete at different curing ages carried out by Chen et al. [34]. They also found the dynamic compression stress-strain curves under different strain rates and curing ages experience strain hardening stages to strain softening ones. However, the strain hardening stage was not so obvious with the curing ages (6 h~28 d) and strain rates (35–178 s^−1^) in the literature. From Figure 8, we can also find from the curves that equivalent aging for 2 years and less are close in general under the same impact velocity, implying that short-time aging has small impact on the performance of EPC. The compression stress in cases of equivalent aging for more than 2 years is generally lower than other cases, which indicates that deterioration becomes obvious after aging. 

**Figure 9:**
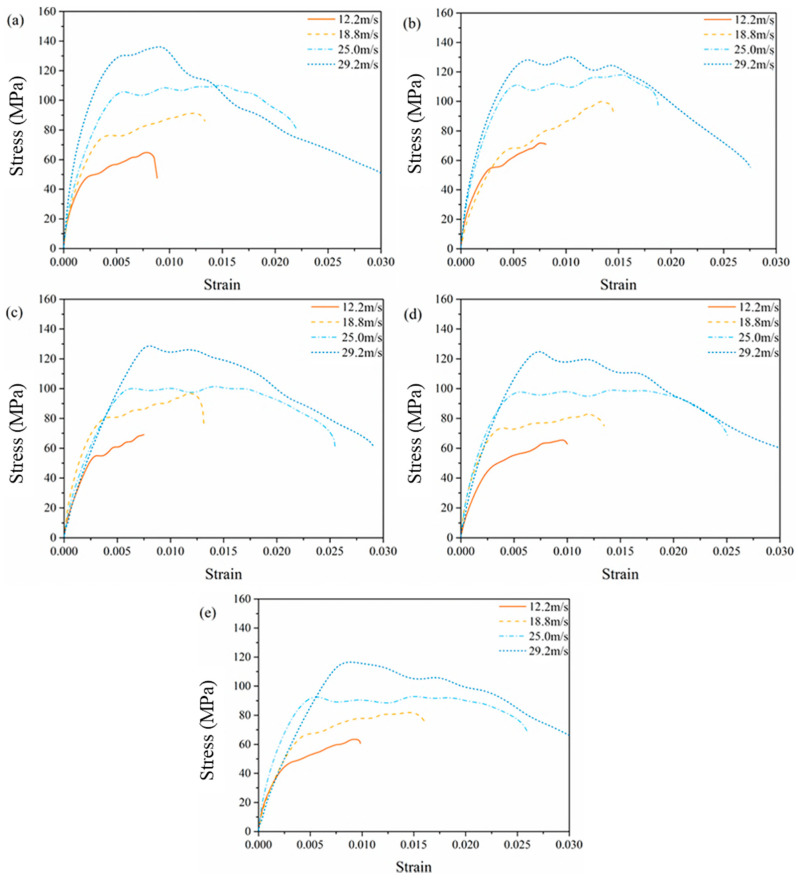
Average compression stress-strain curves with different equivalent aging time: (**a**) 0 years, (**b**) 1 year, (**c**) 2 years, (**d**) 4 years, (**e**) 6 years.

The compression stress-strain curves under different impact velocities and equivalent aging times are shown in Figure 9, and the slope of the curves are listed in Table 3. From this figure, EPC appears to have an obvious strain rate effect with the increase of impact velocity regardless of different aging time. The larger the impact velocity is, the larger the compression stress value corresponding to the transition point between the two stages is. The slope of linear segment of the compression stress-strain curves here reflects the rigidity of the specimens under impact loading. For the case of the blank control group, the slope of the first stage increases with the increase of the impact velocity. However, with the increase of the equivalent aging time, the difference between slopes decreases and the relative position of the first stage of the curves becomes irregular.

**Figure 10:**
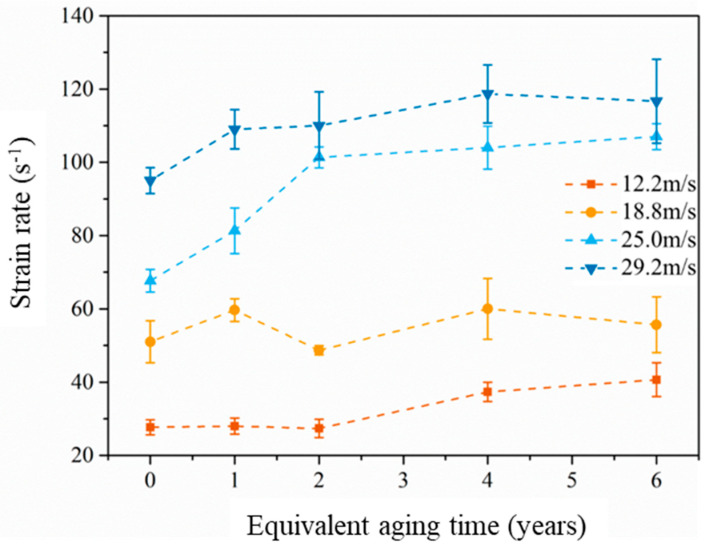
Strain rate-equivalent aging time curves with different impact velocities.

The strain rate under different impact velocities and equivalent aging times is shown in Figure 10 and listed in Table 4. While the aging time varies from 0 years to 6 years, the strain rate grows from 27.7 s^−1^ to 40.7 s^−1^ under the same impact velocity 12.2 m/s; the strain rate grows from 95.0 s^−1^ to 116.7 s^−1^ in the case of 29.2 m/s. It seems that the aging procedure makes EPC turn soft and increases the strain rate. For the case of unsaturated polyester polymer concrete (UPPC) in Reference [34], with the curing ages 6 h, 3d, 7d and 28 d, the strain rate achieved 35 s^−1^, 35 s^−1^, 22 s^−1^ and 42 s^−1^, respectively under the impact velocity 6m/s; and the strain rate achieved 161 s^−1^, 178 s^−1^, 173 s^−1^ and 142 s^−1^, respectively, under the impact velocity 17 m/s. It seems that curing age does not obviously affect the strain rate of UPPC under the same impact velocity.

**Figure 11:**
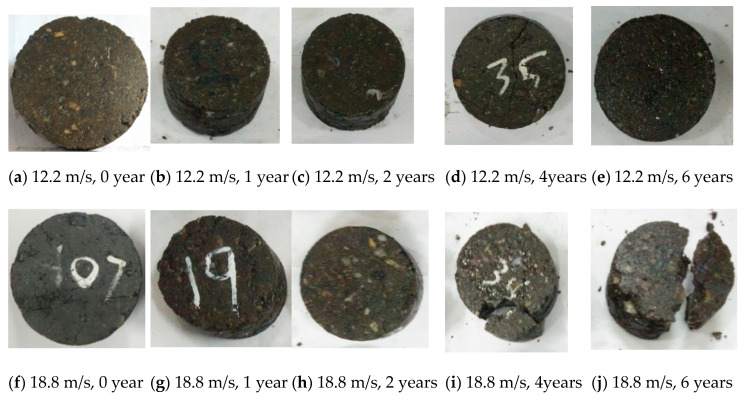

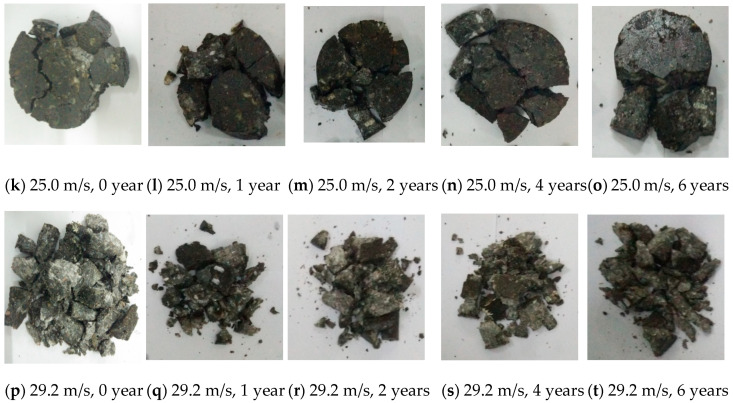
Failure phenomenon under different impact velocities and equivalent aging time.

The failure phenomenon under different impact velocities and equivalent aging times is shown in Figure 11. The label “12.2 m/s, 0 year” means the specimen was impacted under a 12 m/s velocity and aged for 0 years. From the apparent phenomenon of the specimens with different aging times after the 12.2 m/s impact, except for an obvious main crack in the case of aging of 4 years, no obvious damage phenomenon was found in the other specimens. It seems that the internal damage of the specimens after aging for 4 years or more has already existed, and it is within the critical damage range of cracking. This conclusion can also be confirmed by the failure of the specimen under the higher impact velocities below. When the impact velocity was 18 m/s, micro cracks generated in the specimens after a shorter aging time less than 2 years, as shown in Figure 11f–h, and yet the specimens underwent longer aging time not less than 4 years were broken into large pieces, as shown in Figure 11i,j. When the impact velocity increased to 25.0 m/s, the specimens were all broken into large pieces, as shown in Figure 11k–o. When the impact velocity reached 29.2 m/s, the specimens were broken into small pieces, as shown in Figure 11p–t.

### 3.3. Strain Rate Sensitivity 

**Figure 12:**
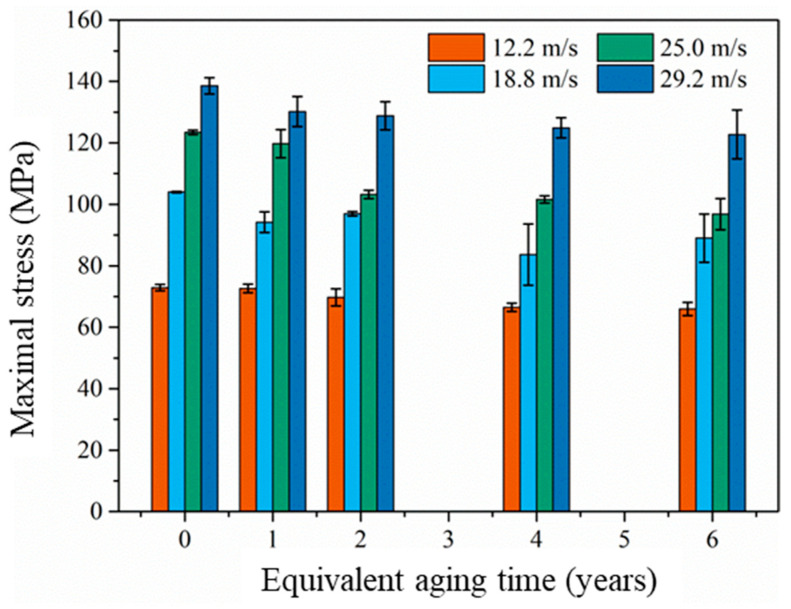
Maximal compression stress under different impact velocity with different equivalent aging time.

The maximal compression stress under different impact velocity with different equivalent aging times is shown in Figure 12 and listed in Table 5. It can be found that the maximal compression stress increases with the increase of impact velocity after aging for the same time, and the maximal compression stress decreases with the increase of the equivalent aging time under the same impact velocity. The correlation coefficient between the maximal compression stress and impact velocity after aging for 0 years, 1 year, 2 years, 4 years and 6 years are, respectively, 0.996, 0.997, 0.968, 0.985 and 0.969. This indicates that the maximal compression stress of EPC is evidently positively correlated with the impact velocity. The correlation coefficient between the maximal compression stress and equivalent aging time with impact velocities 12.2 m/s, 18.8 m/s, 25.0 m/s and 29.2 m/s are, respectively, −0.958, −0.798, −0.904 and −0.914, implying that the maximal compression stress of EPC is negatively correlated with equivalent aging time. 

**Figure 13:**
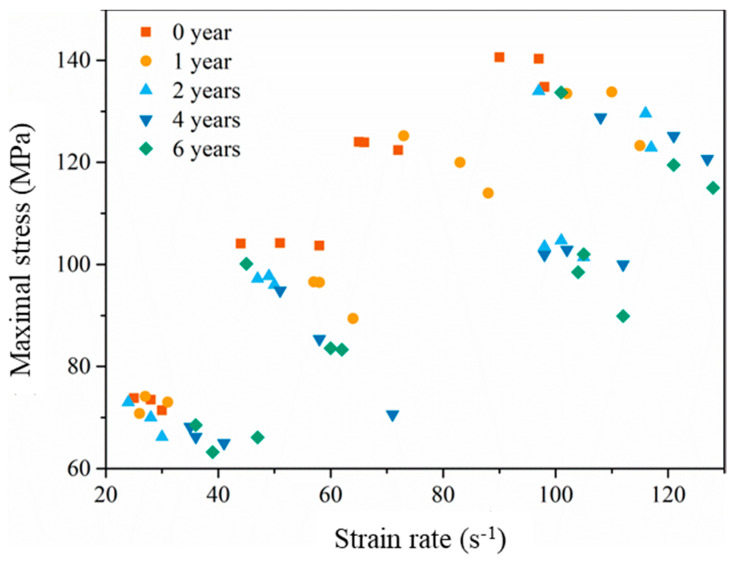
The scatter of the maximal compression stress with different strain rate.

The scatter of the maximal compression stress with different strain rates is shown in Figure 13. It can be observed that the maximal compression stress is sensitive to the strain rate in all aging cases. When the strain rate is 59 s^−1^, the maximal stress decreased from 103 MPa to 84 MPa with the equivalent aging time increasing from 0 to 6 years. For the case of the strain rate 99 s^−1^, the maximal compression stress decreased from 140 MPa to 134 MPa after 6-year equivalent aging. According to the unsaturated polyester polymer concrete in Chen’s work [34], the polymer concrete also appears to have an obvious strain rate strengthening effect, and the maximal compression stress varied from 82.7 MPa to 107.4MPa while the strain rate grows from 35 s^−1^ to 161 s^−1^ after 6 h curing, 96.4 MPa to 141.7 MPa while the strain rate grows from 35 s^−1^ to 178 s^−1^ after 3 d curing, 103.6 MPa to 143.2 MPa while the strain rate grows from 22 s^−1^ to 173 s^−1^ after 7 d curing, and 105.8 MPa to 128.9 MPa while the strain rate grows from 42 s^−1^ to 142 s^−1^ after 28 d curing. It is obvious that the maximal compression stress increased monotonously with the increase of the strain rate, and the maximal compression stress increased with the increase of the curing age at a similar strain rate. Compared with the experimental results of the similar work [34,35,36], it can be confirmed that the accelerated aging environment mentioned in this study can indeed weaken the mechanical properties of epoxy polymer concrete. 

Dynamic increase factor (DIF) is introduced to characterize the increase of the maximal compression stress with the increase of strain rate.

(9) $$DIF=\frac{\sigma_{d}}{\sigma_{s}}$$

where $\sigma_{s}$ is the static strength and $\sigma_{d}$ is the dynamic maximal compression stress. When analyzing the dynamic properties of concrete by an SHPB device, the increase rate of the dynamic strength is often evaluated by the relationship between DIF and the logarithm of strain rate [29,30,31,32,34,35,36,37].

**Figure 14:**
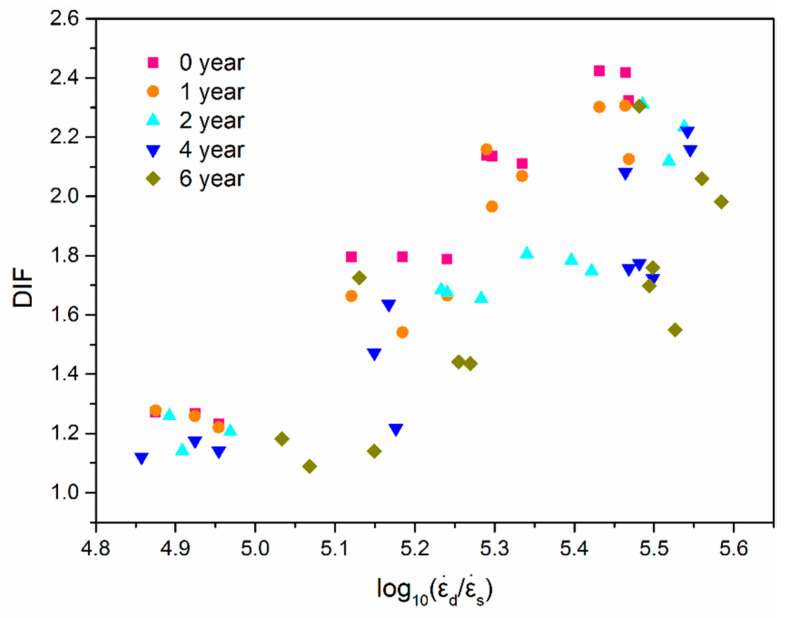
The scatter of the DIF with $\log_{10}({\dot{\varepsilon }}_{d}/{\dot{\varepsilon }}_{s})$.

The strengthening effect of strain rate is shown in Figure 14. The x-axis is $\log_{10}(\frac{{\dot{\varepsilon }}_{d}}{{\dot{\varepsilon }}_{s}})$, where ${\dot{\varepsilon }}_{s}$ is the strain rate of quasi-static compression, and ${\dot{\varepsilon }}_{d}$ is the strain rate in dynamic compressive tests. It is obvious that EPC shows a strain rate strengthening effect, that is, DIF increases with the increase of strain rate. However, the longer the aging time is, the smaller the increase of DIF is, which indicates that UV radiation aging weakens the dynamic strengthening effect of EPC. When the strain rate is 59 s^−1^, the DIF of EPC without aging decreased from 1.78 to 1.44 with the equivalent aging time increasing from 0 to 6 years. For the case of the strain rate 99 s^−1^, the DIF decreased from 2.42 to 2.30 after 6-years equivalent aging.

In order to investigate the importance of strain rate and the equivalent aging time on the results of the maximal compression stress, an ANOVA analysis was carried out with the experimental results. The F-value and the P-value are as follows: (1) the maximal compression stress and strain rate, F-value is 129.8, and P-value is $3.43\times 10^{-25}$; (2) the maximal compression stress and the equivalent aging time, F-value is 12.1, and P-value is $7.63\times 10^{-10}$; (3) the strain rate with the equivalent aging time, F-value is 36.7, and P-value is $6.23\times 10^{-15}$. Obviously, all the P-values are far less than 0.05; the results were statistically significant. The maximal compression stress strongly depended on the strain rate and the equivalent aging time, and the strain rate also depended on the equivalent aging time.

**Figure 15:**
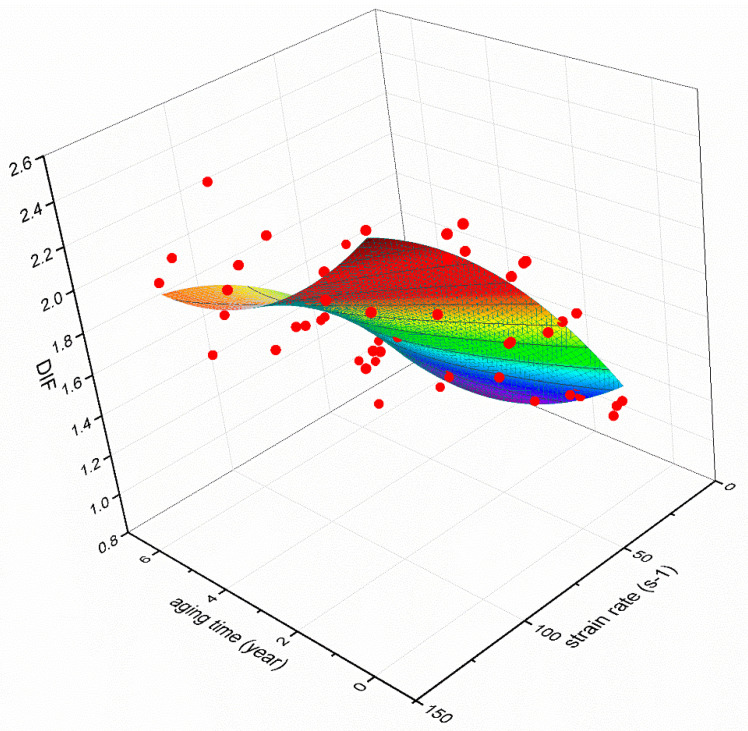
Variation of DIF with strain rate and the equivalent aging time.

To further predict the effect of strain rate and equivalent aging time on DIF, the experimental data points are marked on a three-dimensional coordinate and a spatial surface fitting is carried out. Figure 15 shows the variation of DIF with strain rate and the equivalent aging time. The surface function is as follows:

(10) $$\begin{array}{lll} DIF & = & -6.34\times 10^{-5}{\dot{\varepsilon }}^{2}+0.02\dot{\varepsilon }-5.18\times 10^{-4}\dot{\varepsilon }t-0.15t+0.02t^{2}+0.861\\ R^{2} & = & 0.78 \end{array}$$

where *t*, (y), is the equivalent aging time. Obviously DIF is greatly influenced by strain rate; when subjected to a given strain rate, the longer the aging time is, the smaller the DIF of EPC is. 

## 4. Conclusions

The dynamic mechanical properties of EPC after experiencing different durations of accelerated aging were studied using an SHPB system. Through the experimental study and mechanism analysis, the following conclusions can be obtained:

1. In accordance with the meteorological statistics of Guangzhou city, an accelerated aging procedure, including ultraviolet radiation, temperature and humidity change, were designed to simulate the outdoor environment in South China. The EPC specimens subjected to accelerated aging for 93.3 h is equivalent to natural aging for 1 year.
2. Theoretical analysis was carried out to verify the assumption of stress uniformity in the SHPB test. In this study, the loading time of the incident wave is about 270 μs, the time when failure occurred was about 200 μs after the first reflection, much longer than the required 55 μs to achieve stress uniformity. The specimen has enough time to achieve the stress uniformity before failure, which ensures the validity of the experimental results.
3. The experimental results in the same group under the same impact velocity and aging time have good repeatability. All the compression stress-strain curves show obvious two-stage form regardless of aging time. With the increase of impact velocity, the curves transform from typical strain hardening to strain softening, and there exists a transforming impact velocity of about 25.0 m/s.
4. The EPC appears to have an obvious strain rate effect with the increase of impact velocity for all aging time cases. The larger the impact velocity is, the larger the compression stress value corresponding to the transition point between the two stages. The maximal compression stress increases with the increase of impact velocity after aging for the same amount of time. The maximal compression stress decreases with the increase of equivalent aging time under the same impact velocity, and the aging procedure makes EPC turn soft and increases the strain rate.
5. The failure phenomenon of EPC under different impact velocities and equivalent aging time shows that the failure mode is mainly determined by the impact speed. At the same impact speed, the longer the aging time is, the more easily the specimen is damaged.
6. The variation of DIF with strain rate and equivalent aging are predicted by a fitting curved surface function. DIF is greatly influenced by strain rate; when subjected to a given strain rate, the longer the aging time is, the smaller the DIF of EPC is.

## Figures and Tables

**Table 1:** Parameters of the Specimen and Bar.

Property	Specimen	Bar
Density (kg/m^3^)	2154	7850
Elastic compression modulus (GPa)	30	210

**Table 2:** Relationship between impact velocity and trigger pressure.

**Trigger pressure (MPa)**	0.2	0.5	0.9	1.2
**Impact velocity (m/s)**	12.2	18.8	25.0	29.2

**Table 3:** The slope of the compression stress-strain curves under different impact velocity and equivalent aging time.

Equivalent Aging Time (years)	Impact Velocity (m/s)	Slope (GPa)
0	12.2	43.00 ± 10.06
	18.8	50.78 ± 13.44
	25	50.54 ± 5.14
	29.2	79.40 ± 29.73
1	12.2	29.50 ± 12.28
	18.8	55.60 ± 7.46
	25	61.47 ± 3.80
	29.2	70.33 ± 11.29
2	12.2	43.13 ± 13.32
	18.8	54.37 ± 15.76
	25	39.07 ± 15.44
	29.2	28.67 ± 3.61
4	12.2	24.87 ± 5.42
	18.8	53.30 ± 7.24
	25	52.17 ± 4.56
	29.2	37.30 ± 4.12
6	12.2	31.57 ± 8.00
	18.8	28.93 ± 10.43
	25	48.57 ± 7.22
	29.2	24.43 ± 5.17

**Table 4:** The strain rate under different impact velocity and equivalent aging time.

Equivalent Aging Time (years)	Impact Velocity (m/s)	Strain Rate (s^−1^)
0	12.2	27.62 ± 2.05
	18.8	51.08 ± 5.72
	25.0	67.57 ± 3.09
	29.2	95.10 ± 3.56
1	12.2	28.11 ± 2.16
	18.8	59.60 ± 3.09
	25.0	81.37 ± 6.24
	29.2	109.00 ± 5.35
2	12.2	27.38 ± 2.49
	18.8	48.81 ± 1.25
	25.0	101.33 ± 2.87
	29.2	110.04 ± 9.20
4	12.2	37.30 ± 2.62
	18.8	60.06 ± 8.29
	25.0	104.11 ± 5.89
	29.2	118.67 ± 7.93
6	12.2	40.65 ± 4.64
	18.8	55.59 ± 7.59
	25.0	107.01 ± 3.56
	29.2	116.81 ± 11.44

**Table 5:** The maximal compression stress under different impact velocity and equivalent aging time.

Equivalent Aging Time (years)	Impact Velocity (m/s)	Maximal Compression Stress (MPa)
0	12.2	72.90 ± 1.07
	18.8	104.00 ± 0.22
	25.0	123.43 ± 0.73
	29.2	138.57 ± 2.67
1	12.2	72.63 ± 1.37
	18.8	94.17 ± 3.37
	25.0	119.73 ± 4.58
	29.2	130.20 ± 4.88
2	12.2	69.73 ± 2.78
	18.8	96.97 ± 0.71
	25.0	103.20 ± 1.36
	29.2	128.83 ± 4.56
4	12.2	66.47 ± 1.32
	18.8	83.63 ± 10.00
	25.0	101.60 ± 1.20
	29.2	124.90 ± 3.31
6	12.2	65.93 ± 2.17
	18.8	89.00 ± 7.84
	25.0	96.80 ± 5.08
	29.2	122.73 ± 7.97
